# Maternal Neurofascin-Specific Autoantibodies Bind to Structures of the Fetal Nervous System during Pregnancy, but Have No Long Term Effect on Development in the Rat

**DOI:** 10.1371/journal.pone.0085393

**Published:** 2014-01-20

**Authors:** Sonja Hochmeister, Thomas Pekar, Maren Lindner, Maja Kitic, Michaela Haindl, Maria Storch, Franz Fazekas, Christopher Linington

**Affiliations:** 1 Medical University Graz, Department of Neurology, Graz, Austria; 2 Medical University Vienna, Brain Research Institute, Department of Neuroimmunology, Vienna, Austria; 3 University of Glasgow, Institute of Infection, Immunity and Inflammation, Glasgow, United Kingdom; Hospital Nacional de Parapléjicos – SESCAM, Spain

## Abstract

Neurofascin was recently reported as a target for axopathic autoantibodies in patients with multiple sclerosis (MS), a response that will exacerbate axonal pathology and disease severity in an animal model of multiple sclerosis. As transplacental transfer of maternal autoantibodies can permanently damage the developing nervous system we investigated whether intrauterine exposure to this neurofascin-specific response had any detrimental effect on white matter tract development. To address this question we intravenously injected pregnant rats with either a pathogenic anti-neurofascin monoclonal antibody or an appropriate isotype control on days 15 and 18 of pregnancy, respectively, to mimic the physiological concentration of maternal antibodies in the circulation of the fetus towards the end of pregnancy. Pups were monitored daily with respect to litter size, birth weight, growth and motor development. Histological studies were performed on E20 embryos and pups sacrificed on days 2, 10, 21, 32 and 45 days post partum. Results: Immunohistochemistry for light and confocal microscopy confirmed passively transferred anti-neurofascin antibody had crossed the placenta to bind to distinct structures in the developing cortex and cerebellum. However, this did not result in any significant differences in litter size, birth weight, or general physical development between litters from control mothers or those treated with the neurofascin-specific antibody. Histological analysis also failed to identify any neuronal or white matter tract abnormalities induced by the neurofascin-specific antibody. Conclusions: We show that transplacental transfer of circulating anti-neurofascin antibodies can occur and targets specific structures in the CNS of the developing fetus. However, this did not result in any pre- or post-natal abnormalities in the offspring of the treated mothers. These results assure that even if anti-neurofascin responses are detected in pregnant women with multiple sclerosis these are unlikely to have a negative effect on their children.

## Introduction

Neurofascin (Nfasc) is a cell adhesion molecule belonging to the immunoglobulin superfamily (IgSF). Several neurofascin isoforms are generated by alternative splicing (155 kDA, 166, 180 and 186 kDa) and their expression is temporally and spatially regulated during development in the central [1]–[4] and peripheral nervous system [5], [6]. Neuronal neurofascin (Nfasc186) is localized at the nodes of Ranvier and axonal initial segments (AIS) of myelinated fibers where it interacts with voltage gated sodium channels and other proteins such as ankyrin G and ß IV-Spectrin [7], [8]. In contrast, neurofascin-155 (Nfasc155) is an oligodendroglial product [9] sequestered in septate-like junctions where the paranodal loops of the myelin sheath contact the axonal surface. Nfasc155 interacts with the axonal Caspr-Contactin complex at these sites [10] to form electron dense assemblies characteristic of this paranodal junction complex. These complexes play a critical role in maintaining saltatory conduction by physically separating NaV1.6 channels at the node from K_v_1.1 and 1.2 potassium channels located within the juxtaparanodal domain of the axolemma [11], [12].

Apart from the voltage gated sodium channel the neurofascins remain the only proteins known to be essential for nodal assembly and saltatory conduction in the central nervous system [13], [14]. It therefore not surprising that perturbation of neurofascin expression has dramatic pathophysiological consequences [11]. Neurofascin- null mice exhibit severe ataxia, motor paresis and severe reduction of nerve conduction velocities and have a dramatically reduced life span of only 3 weeks [13], [15]. They neither form paranodal adhesion junctions nor nodal complexes [11]. Selective genetic ablation of Nfasc186 during development results in nodal disorganization, including loss of Na(v) channel and ankyrin-G (AnkG) enrichment at nodes [12], as well as neuron degeneration and severe ataxia [16]. After completion of development, neurofascin is believed to anchor key elements of the adult AIS complex [14]. Loss of neurofascin expression by adult neurons leads to slow disorganization of the AIS and pinceau morphology [16] with consequent impairment of motor learning and abolition of the spontaneous tonic discharge typical of purkinje cells [14]. Similarly, ablation of glial Nfasc155 in adult myelinating glia leads to a gradual disorganization of paranodal axoglial junctions as the levels of neurofascin protein at the paranodes decline [15]. Changes in the distribution of neurofascin isoforms in the nodal domains of myelinated axons are also seen in multiple sclerosis (MS) lesions [1], [17].

Neurofascin is also a target for autoantibodies, as demonstrated by the presence of neurofascin-specific autoantibodies in patients with MS [18], [19], Guillain-Barre syndrome [20], and chronic idiopathic demyelinating neuropathy [21], [22]. In an animal model of multiple sclerosis these antibodies were shown to aggravate disease severity by disrupting axonal conduction, triggering axonal injury and inhibiting remyelination [18], [23]–[25].

Previous studies in myasthenia gravis and systemic lupus erythematosus demonstrate transplacental exposure to maternal autoantibody can mediate severe effects on the developing fetus [26]–[28]. Given the diverse and vital role of neurofascin during development of the central and the peripheral nervous system we speculated anti-neurofascin antibodies might have negative effects on fetal development. Although there is no obvious clinical evidence for increased negative outcome of pregnancies in mothers with MS subtle differences might have gone undetected thus far. In our study we sought to address this question by passively immunizing pregnant rats with a pan-neurofascin antibody derived from the mouse.

## Materials and Methods

### Antibody generation

A12/18.1 hybridoma cells secreting a pan-Nfasc monoclonal mouse IgG2a antibody (as described in [18]) were cultured in CELLine® cell culture flasks (BD biosciences) following manufacturer's guidelines. The media compartment was filled with 200 ml complete DMEM containing 10% fetal calf serum, 2 mM L-glutamine (200 mM), 1 mM sodium pyruvate, 50 mM ß-mercaptoethanol, 1% non-essential amino acids and 1% penicillin/streptomycin and the cell compartment inoculated with 2×10^6^ cells/ml diluted in complete DMEM. Supernatant was harvested from the cell compartment after 7 days and twice a week thereafter. Supernatants were centrifuged and stored at −20°C. Antibody production was monitored by ELISA and mAb purified from pooled supernatants by protein G chromatography.

### Animals

Twenty 8–10 weeks old pregnant Sprague Dawley rats obtained from Versuchstierzuchtanstalt Himberg/Medical University of Vienna and were housed in the animal facility of the Institute for Biomedical Research/Medical University Graz in groups of two rats/cage with access to food and water at libitum. On days E15 and E18 of pregnancy rats were intravenously injected with either 1 mg anti-neurofascin monoclonal antibody (10 rats; antibody generated as described above) or the same dose of a commercially available mouse IgG2a myeloma protein (8 rats, Sigma- Aldrich, St.Louis, MO) as an isotype control not directed against any central nervous system antigen. One pregnant rat received 0,5 mg anti-neurofascin antibody on days E15 and E18, respectively; another pregnant rat did not undergo any treatment and its offspring were used as controls for weight and motor development.

For the injections the rats were briefly anaesthetized with isoflurane. On day E20, two rats of the neurofascin antibody treated group and one of the control antibody group were deeply anesthetized with isoflurane and sacrificed by cervical dislocation for histological analysis of the pups. The pups were sacrificed by decapitation. The remaining pregnant rats were allowed to give birth to their offspring; these were monitored by daily observation by two blinded investigators with respect to litter size, birth weight, growth curve and motor development up to day 32. For histological analysis of the fully mature rat central nervous system, some rats were kept until 45 days of age. In total, 236 pups were analysed in this experiment. The number of pups and their respective age at sacrifice and subsequent histological analysis is shown in Table 1. The animal experiment was approved by the Austrian ministry of Science and Research (GZ 66.010/0056-II/10b/2010). All efforts were made to minimize suffering of the animals.

**Table 1:** Number of pups analysed with respect of developmental stage.

	E20	p2	p10	p15	p21	p32	p42
**anti-neurofascin antibody 2×1mg**	27	20	25	31	20	15	15
**anti neurofascin antibody 2×0,5mg**	0	4	5	4	3	0	3
**isotype matched control antibody 2×1mg**	7	11	5	5	5	14	5
**untreated control**						12	
**pups total**	34	35	35	40	28	41	23

E20 means day 20 of gestation, p2, p10, p15, p21, p32, p42 represents the respective age in days (postnatal days) at time of sacrifice.

### Tissue processing and immunohistochemistry

On days E20 and postnatal day(p) p2, p10, p21, p32 and p45 groups of pups were sacrificed; E20 and p2 pups were decapitated, p10, p21, p32 and p45 pups were deeply anesthetized with isoflurane, sacrificed through cervical dislocation and then perfused transcardially with 4% PFA. Brains and spinal cords were postfixed in 4%PFA over 24 hours and embedded in paraffin. Serial sections of 2–3μm were cut using a microtome. After deparaffinization in XEM-200 for 20 min (Vogel GmbH, Giessen, Germany) and washing in alcohol solutions of decreasing concentrations (from 96%/70%/50% to deionized water). Sections were stained with hematoxylin/eosin and luxol fast blue to assess gross developmental abnormalities.

In adjacent serial sections immunohistochemistry was performed according to the following protocol: Antigen retrieval was performed by heating slides in citrate buffer at pH 6.0 for one hour in a commercial food steamer. After cooling and washing in phosphate buffered saline (PBS), slides were incubated over night with antibodies either directed against neurofascin (generated as described above) at a dilution of 1∶50 or anti-mouse IgG (GE Healthcare, Buckinghamshire, UK) at a dilution of 1∶200. The antibodies were diluted in 10% fetal calf serum/PBS. Bound primary antibody was detected with a biotin–avidin technique using 3,3′-diaminobenzidine tetra-hydrochloride (Sigma, St. Louis, MA) as chromogen as previously described in detail [29]. Control sections were incubated in the absence of primary antibody.

Furthermore double staining for neurofascin/mouse IgG was performed for light microscopy.

Neurofascin immunoreactivity (IR) was visualized using a biotin-avidin technique as described above with diaminobenzidine enhanced by Nickel/cobalt as chromogen. The second primary antibody was detected with the biotin-avidin technique using 3-amino-9-ethyl-carbazole (Sigma, St. Louis, MO) as chromogen.

### Confocal laser microscopy

For confocal microscopy, anti-neurofascin antibody was applied at a dilution of 1∶100 at 4°C in a wet chamber over night. After washing with phosphate buffered saline (PBS) biotinylated anti-mouse IgG (GE Healthcare, Buckinghamshire, UK) was applied for 1 hour at room temperature at a dilution of 1∶200, followed by Avidin HRP (1∶100; Sigma, St. Louis, USA) for one 1 hour. After enhancement with 0,1% CSA in PBS/0.03% H_2_O_2_ for 20 min at room temperature (protocol as described in [30]) signal was visualized by Avidin-Cy2 (Jackson ImmunoResearch, West Grove, PA, USA) at 1∶75 for one hour at room temperature. For detection of bound anti-neurofascin antibody in fetal tissue slides were subjected to the protocol as described above but without applying the anti- neurofascin antibody in the first step. Fluorescent preparations were examined using a Leica TCS SP5 laser scanning microscope with LAS AF software (Leica Microsystems, CMS GmbH, Germany). An argon laser was used for detection of Cy2 (488 nm excitation) signal.

### Accelerated rotarod performance test

The rotarod test is a common test to assess motor coordination and balance alterations in the rodent. At age p16, p19, p24, p25 and p32, rats of all three groups (neurofascin treated, control antibody treated and untreated control) were placed on a commercially available rotarod device (IITC Life Sciences Series 8, www.iitcinc.com), consisting of a rotating rod (diameter 3 ¾ inch) situated 20 cm high above a tilting plank. Before the beginning of the sessions, all animals were allowed to habituate with the apparatus during 30 s with no rotation. Then, the speed of rotation gradually increased from 0 to 30 rpm and the time until falling off the rod was measured. Rats were tested three times in each session and the best performance of each rat was collected.

### Grip strength test

To assess the fore limb grip strength of rats of all three experimental groups were placed on a commercially available Grip strength meter (TSE Systems, www.tse-systems.com) on days p24, p25 and p32. In brief, a grid is connected to a sensor. The rats are held by the tail and lowered over the top of the grid so that only their front paws can grip the grid. The animals cling to the mesh by a natural reflex. The torso of the rat is then held horizontal and pulled back steadily by the observer until the animal releases the grid. The maximal grip strength value is measured. Each animal was tested three times at each session and the best value of each session was collected.

### Statistical analysis

Litter size, weight data and motor test results are expressed as mean +− SD. Differences between the groups were assessed pair wise by a parametric univariate t-test. The analyses were performed using Statgraphic software (www.statgraphics.com). The level of p<0.05 was considered significant.

## Results

### Maternal antibody transfer does not alter fetal viability

In total, 236 pups were analysed in this experiment. The average number of pups/litter was 12 +/− 3 pups in the group receiving anti- neurofascin antibody and 11+/− 3 pups/litter in the control group (*p* = 0,33671), ruling out significant fetal loss due to the presence of the anti-neurofascin antibody in the mothers' circulatory system. Birth weight averaged between 4-6 g in both groups, representing a normal rat birth weight. Sex ratio was 1∶ 1,5 (male:female) in the neurofascin antibody treated group, 1∶1,33 in the control antibody group and 1∶ 1,4 in the control group receiving no treatment at all.

### Neurofascin antibodies pass the placental border and reach the fetal central nervous system

In order to see whether intravenously applied antibody actually reached the central nervous system (CNS) of the developing fetus, immunohistochemistry for mouse IgG was performed on pups sacrificed at E20 of pregnancy. This day of sacrifice was chosen to assure a high passively transferred antibody titer present in the mothers' circulation 48 hours after receiving the second dose of the anti-neurofascin antibody or the control antibody, respectively.

**Figure 1:**
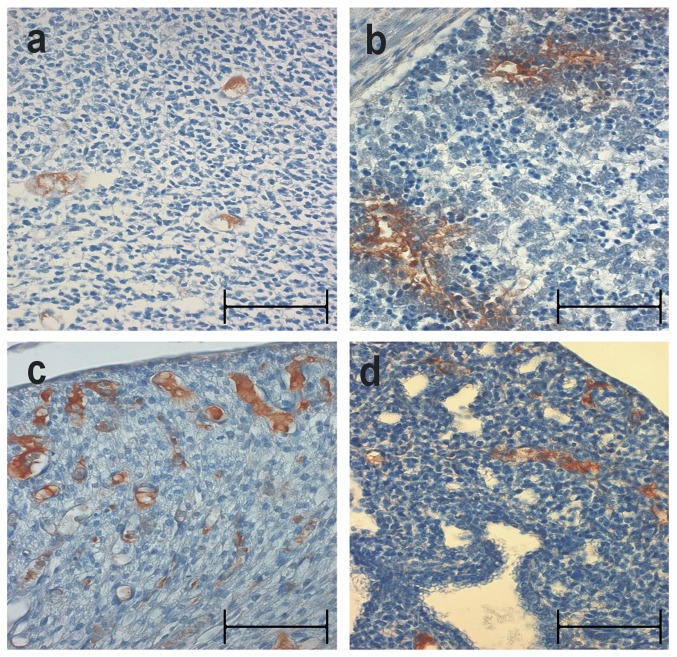
Neurofascin antibodies pass the placental border and reach fetal tissues. Histological analysis of pups (developmental stage E20) 48 hours after application of the second dose of passive antibody transfer. Immunohistochemistry for mouse-immunoglobulin proves antibody transfer into fetal tissue. (a) shows mouse immunoglobulin reactivity in brain tissue vessels of the unborn rat pups, (b) in fetal liver, (c) in the kidney and (d) in the lung of the pups (original magnification 200×). Scale bars represent 50 µm.

Strong immunoreactivity for mouse IgG was seen in blood vessels in the fetal brain, lung, kidney and liver (Fig. 1a, b,c d, respectively) proving that the antibody had crossed the placental border and reached the fetal circulation. Negative control slides (incubated in absence of the primary antibody) showed total absence of staining (not shown).

### Neurofascin staining pattern is not altered by transplacental exposure to neurofascin antibodies

**Figure 2:**
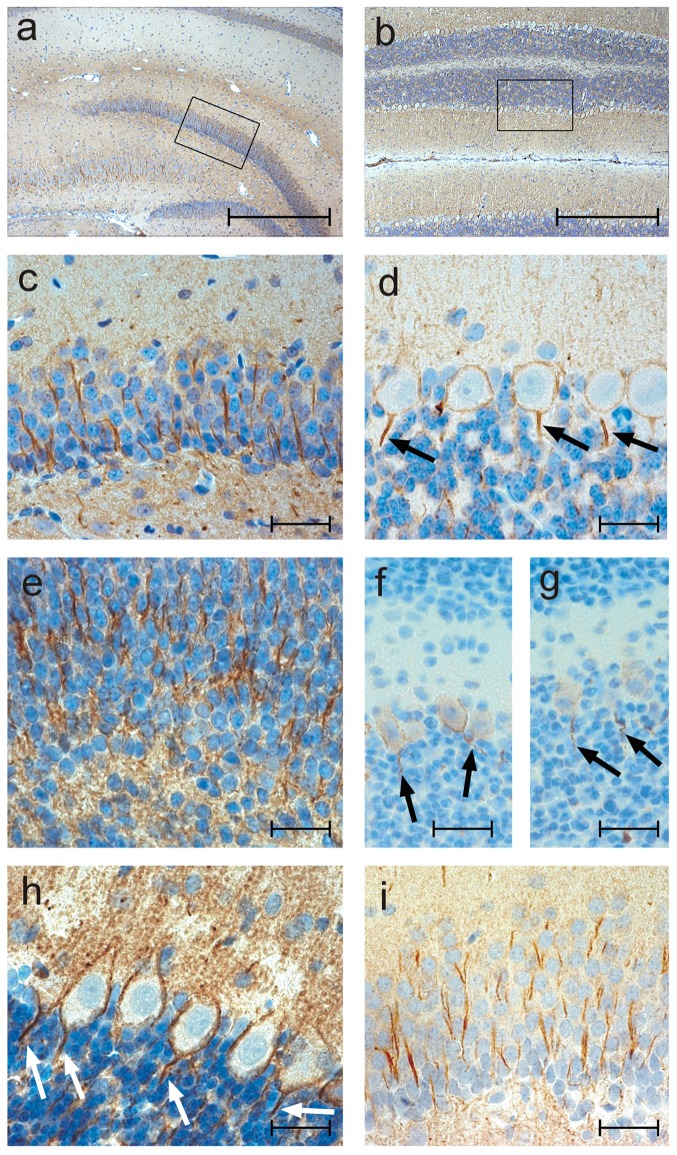
Neurofascin immunoreactivity is not altered by intrauterine exposure to anti- neurofascin autoantibodies. Immunohistochemistry for neurofascin in the normal adult rat brain (a-d) and at different stages of development after intrauterine exposure to the anti- neurofascin antibody (e-i). Areas marked with a rectangle in Fig. 2a and b are shown at higher magnification in c and d, respectively. (a, c) shows immunoreactivity for neurofascin in axons in the hippocampus region, (b, d) in purkinje cells of the cerebellum of normal, untreated adult rats. (e) shows neurofascin staining in the hippocampus region in rats at E20, (f, g) in purkinje cells at postnatal day 10, (h) in purkinje cells at p 21 and (i) in axons of the hippocampus at p21. The arrows in 2d, f-h show the specific neurofascin immunoreactivity of the initial axonal segments of the purkinje cells. (original magnification in a, b 100×, in c-i 630×). Scale bars in a, b represents 100 µm, in c-i 20 µm.

In normal, untreated adult brain tissue, neurofascin immunoreactivity is most pronounced in axons of the hippocampus region (Fig. 2 a, c) and in purkinje cells and their initial axonal segments (Fig. 2 b, d). This staining pattern remains unchanged in pups exposed to neurofascin antibodies *in utero* (Fig. 2 e–i). Fig. 2e shows neurofascin immunoreactivity in the hippocampus region at E20, Fig. 2f and g in the initial axonal segments of purkinje cells at age p10 in offspring of anti-neurofascin antibody treated mothers. Fig. 2h and i show neurofascin immunoreactivity at postnatal day 21 in purkinje cells and in axons of the hippocampus region of neurofascin anibody treated rats, respectively.

The neurofascin staining pattern of the offspring of the rat mother receiving 2×0,5 mg anti-neurofascin antibody was virtually indistinguishable from those receiving the full dose of anti-neurofascin antibody (data not shown).

### Neurofascin antibodies bind to their target structures in the fetal rat brain

**Figure 3:**
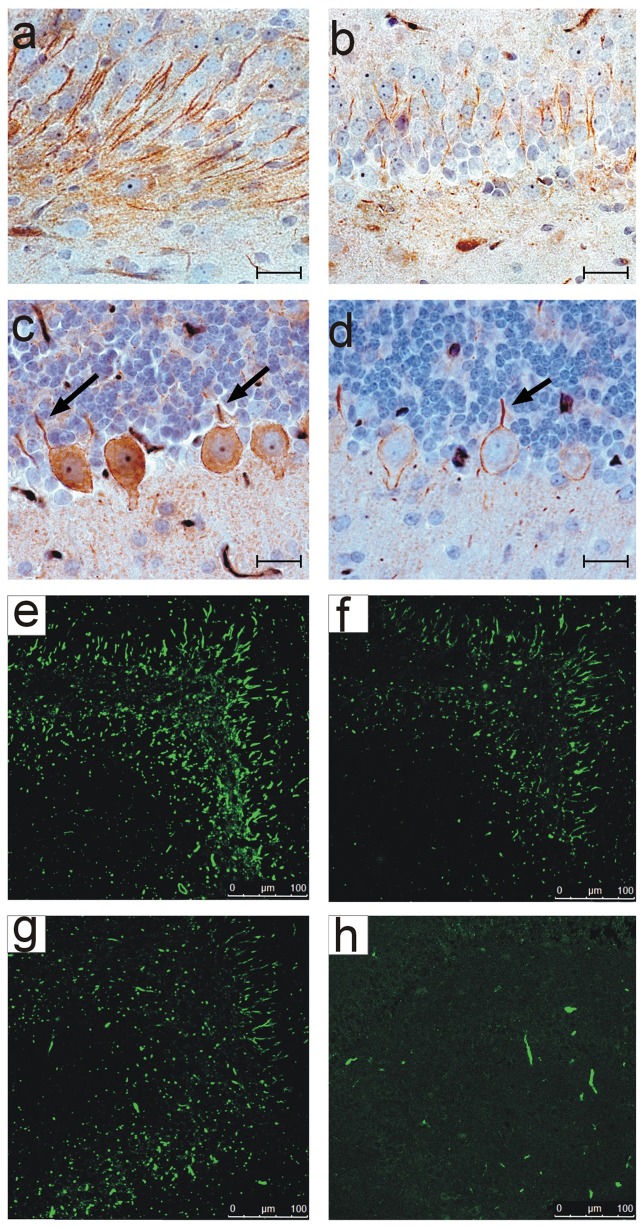
Neurofascin antibodies bind to their target structures in the fetal rat brain. Double stainings for light microscopy for neurofascin (brown) and mouse IgG (red) in rats at p21 are shown in Fig. 3 a-d. In neurofascin antibody treated rats (3a, c) an intense double staining for neurofascin as well as mouse Ig is detectable in axons of the hippocampus region (a) and purkinje cells (c), proving the mouse- derived anti-neurofascin antibody bound to its target structures. Animals treated with control antibody (3b, d) show reactivity for neurofascin in the respective areas, but no staining for mouse immunoglobulin. The black arrows in 3 c, d show the initial axonal segments of the purkinje cells. This staining pattern is confirmed by immunohistochemistry for confocal microscopy of the hippocampus region; staining pattern for neurofascin (e, g) is identical in neurofascin antibody treated animals (e) and control animals (g), but mouse immunoglobulin is only detectable in the neurofascin group (f) but not in the control group (h). Scale bars in a-d represent 20 µm, and in e-h 100 µm.

The total absence of any obvious effect of intrauterine exposure to the anti-neurofascin antibody prompted us to ask the question whether the transferred autoantibody had actually bound to its target in the CNS. We performed immunohistochemistry for combined neurofascin and anti- mouse immunoglobulin for light microscopy on brain tissue of pups from E20 to up to age 21 days. Neurofascin antibody treated pups showed a clear double staining for neurofascin as well as mouse immunoglobulin (Fig. 3a, c), while animals treated with an isotype matched control antibody directed against an CNS irrelevant target revealed the normal neurofascin staining pattern only (Fig. 3 b, d). Single staining for these antibodies for confocal microscopy of the hippocampus region (Fig. 3 e–h) confirmed this staining pattern. The staining pattern for neurofascin is identical in neurofascin antibody treated (e) and control animals (g), but mouse immunoglobulin is only detectable in the neurofascin group (f) but not in the control group (h). Therefore we conclude that the passively transferred antibody bound to its target in the developing CNS, but this alone did not lead to any apparent morphological abnormalities.

### Pup development and motor performance is not altered by transplacental anti-neurofascin antibody transfer

**Figure 4:**
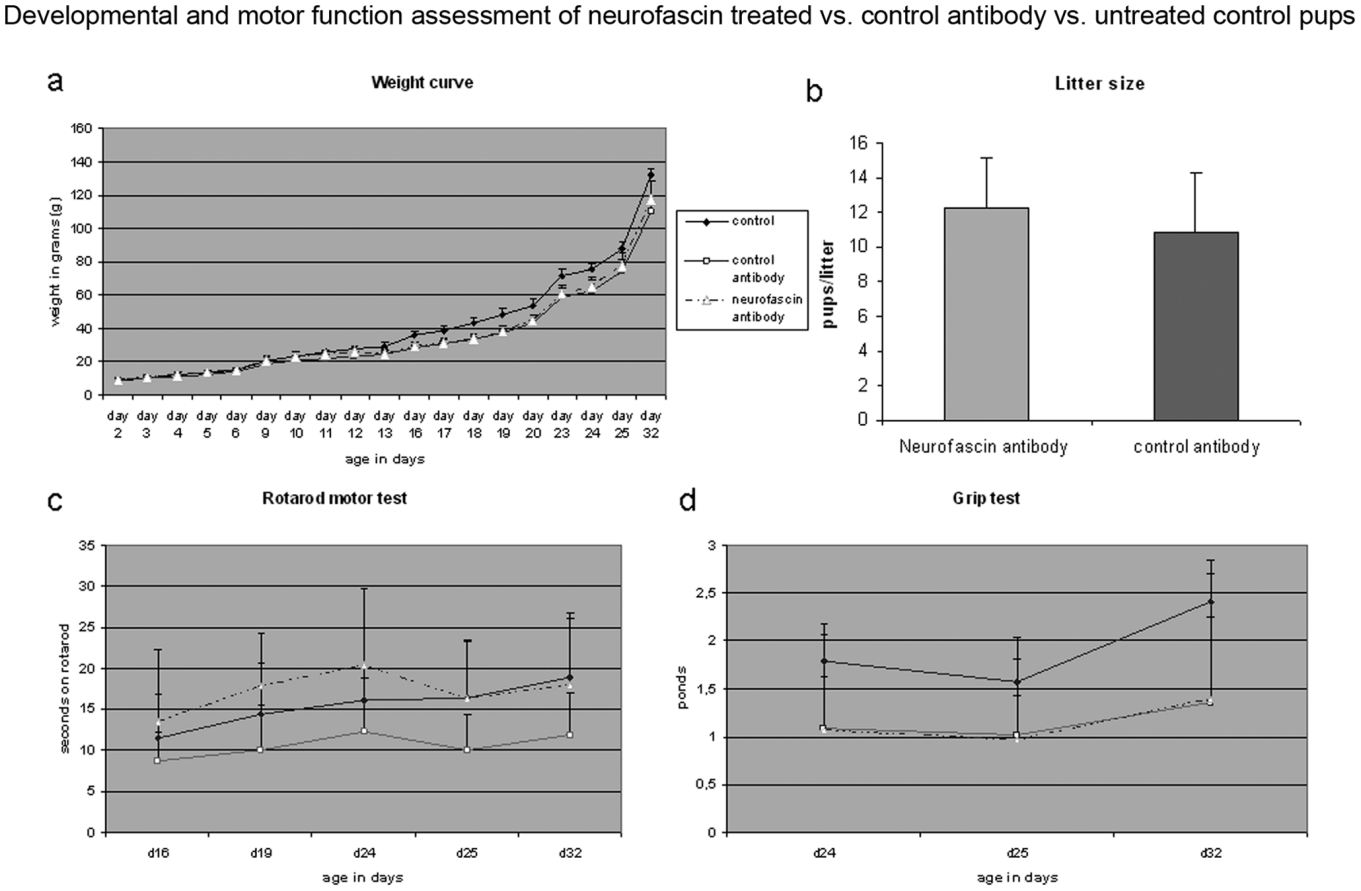
Graphical presentation of vital statistics of neurofascin treated pups vs. control groups. The weight curve from age p2 up to p32 of neurofascin- and control antibody treated pups in comparison to an untreated control rat litter is shown in Fig. 4 a. Weight curves are remarkably similar with no significant differences noted. Also, the litter sizes did not differ significantly between the neurofascin antibody group and the control antibody group (*p* = 0,336712), as shown in Fig. 4 b, ruling out significant fetal loss due to the presence of the antiaxonal antibody. Fig. 4c shows the mean times in seconds +− SD on an accelerating Rotarod device tested on postnatal days 16, 19, 24, 25 and 32. Again, no statistical significant difference between the groups was found (untreated control vs. control antibody group *p* = 0,530163, untreated control vs. neurofascin treatment *p* = 0,354292, control antibody group vs. neurofascin group *p* = 0,117562). Fore limb grip test was performed on postnatal days 24, 25 and 32 and is shown in Fig. 4d. Again, no statistically significant differences were noted (untreated control vs. control antibody group *p* = 0,935596, untreated control vs. neurofascin treatment *p* = 0,429921, control antibody group vs. neurofascin group *p* = 0,593029).

Developing pups were monitored closely by three observers (SH, TP, MH) for up to 6 weeks with respect to growth curve and motor development. Growth curve was unremarkable in both groups and virtually indistinguishable to the developmental curve of fully untreated rat pups (as shown in Fig. 4a). Coordination and balance tested on an accelerating Rotarod device at age p16, p19, p24, p25 and p32 failed to detect any statistical significant differences between the groups (Fig. 4c: untreated control vs. control antibody group *p* = 0,530163, untreated control vs. neurofascin treatment *p* = 0,354292, control antibody group vs. neurofascin group *p* = 0,117562). Similarly, measurement of fore limb grip strength (Fig. 4d) did not reveal any statistical significant differences between groups (untreated control vs. control antibody group *p* = 0,935596, untreated control vs. neurofascin treatment *p* = 0,429921, control antibody group vs. neurofascin group *p* = 0,593029). Histological examination of gross morphology at different developmental stages (E20, p2, p10, p21, p32 and p45) failed to detect any developmental abnormalities caused by exposure to the neurofascin-specific antibody (Fig. 2e–i).

## Discussion

Transplacental transmission of maternal IgG antibodies during fetal development is a physiological process, starting at the end of the first trimester. During the second trimester the antibodies can access the fetal brain resulting in a 50–70 fold increase in IgG in fetal compared with maternal brains [28]. While in healthy mothers this provides perinatal immunity to infectious agents, it is potentially deleterious for children of mothers suffering from autoimmune diseases such as connective tissue disorders, thyroid autoimmune disorders, myasthenia gravis, idiopathic thrombocytopenic purpura (as reviewed in [31], [32]). The time course of clinical symptomatology often parallels the presumed half life of IgG immunoglobulins and often will have no long term effects when the child or mother are treated appropriately. However, this is not necessarily the case as demonstrated in patients with systemic lupus erythematosus (SLE). This disease preferentially affects women in their childbearing years, and in up to 40% of cases is associated with an autoantibody response to double-stranded DNA that can cross-react with NMDA-receptors in the CNS. In a mouse model of the SLE a similar response is associated with reduced female fetal viability [27], an observation consistent with available sex ratio data on live births in women with SLE. Furthermore, children of mothers, but not fathers suffering from SLE have a high incidence of learning disorders [33]–[37]. An observation that led to the demonstration that high titres of DNA/NMDAR-cross reactive autoantibodies in a mouse model of SLE induce histological abnormalities in the developing brain that result in cognitive impairment in adulthood [28].

As Nfasc186 is expressed during CNS development we speculated maternal autoimmunity to neurofascin might also be detrimental for developing fetus. This is not a trivial question as multiple sclerosis is associated with a neurofascin-specific autoantibody response and preferentially affects young women.

In this study, we passively transferred a potentially pathogenic pan-neurofascin IgG2a mAb into pregnant dams on days 15 and 18 post conception to mimic the physiological concentration of maternal antibodies into the fetus towards the end of pregnancy. Our results demonstrate that the passively transferred antibody crossed the placental barrier and bound to sites at which neurofascin is expressed in the CNS. However, this was not sufficient to induce developmental abnormalities as determined by either histopathological examination or clinical observation. We conclude that even if anti-neurofascin responses are detected in pregnant patients these antibodies are unlikely to have a detrimental effect on fetal development.
